# Supplementary material for: Complexome profiling on the Chlamydomonas lpa2 mutant reveals insights into PSII biogenesis and new PSII associated proteins
Source: J Exp Bot. 2021 Aug 26;73(1):245–62. doi: 10.1093/jxb/erab390 (PMC8730698; doi:10.1093/jxb/erab390)
Supplement: erab390_suppl_Supplementary_Dataset_S1 [file erab390_suppl_supplementary_dataset_s1.zip › Supplemental Dataset 1 - Excel List and all profiles/plots/RPS15_Cre08.g360900.html]

Trivial name: RPS15  
  
Euclidean distance: 4963.97  
Mean Intensity (WT): 361.36  
Mean Intensity (Mut): 339.97  
Distance: 13.74  
  
MapMan: protein.synthesis.ribosomal protein.unknown.small subunit.S19;protein.synthesis.ribosomal protein.eukaryotic.40S subunit.S15  
  
p value of intensity sums Welch test: 0.8979
